# Mortality trends and causes of death in a South African hospital complex pre- and during COVID-19

**DOI:** 10.4102/sajid.v40i1.679

**Published:** 2025-03-06

**Authors:** Khanyisile M. Tshabalala, Inger Fabris-Rotelli, Debashis Basu, Magriet Myburgh, Fareed Abdullah

**Affiliations:** 1 Department of Public Health Medicine, School of Health Sciences, Steve Biko Academic Hospital, Tshwane, South Africa; 2 Department of Public Health Medicine, Faculty of Health Sciences, University of Pretoria, Tshwane, South Africa; 3 Department of Statistics, Faculty of Statistics, University of Pretoria, Tshwane, South Africa; 4 Department of Health Information Management, Steve Biko Academic Hospital, Tshwane, South Africa; 5 Department of Infectious Diseases and Public Health Medicine, Faculty of Health Sciences, Steve Biko Academic Hospital, Tshwane, South Africa; 6 Office of AIDS and TB Research, South African Medical Research Council, Tshwane, South Africa

**Keywords:** COVID-19, in-hospital mortality trends, causes of death

## Abstract

**Background:**

Before coronavirus disease 2019 (COVID-19), global health was improving, with declining mortality trends. The pandemic disrupted this progress, increasing mortality in South Africa between April 2020 and March 2022. Pre-pandemic data establishes a baseline for assessing COVID-19’s impact on all-cause mortality.

**Objectives:**

This study examines changes in hospital-based mortality trends in a Gauteng hospital complex from April 2018 to March 2022, addressing the scarcity of such studies during the COVID-19 era.

**Method:**

A retrospective review of 7815 deaths from April 2018 to March 2022 was conducted. Chi-squared tests were used to analyse deaths by age group and gender, with correlations reported.

**Results:**

Mortality rates rose from 3.2% in 2018–2019, peaked at 5.1% in 2020–2021, and declined to 4.2% in 2021–2022. Patients aged 15 years–64 years had the highest death rates, with an increase among those over 65. Male deaths exceeded female deaths, with the smallest difference observed in 2020–2021. Leading causes of death included diseases of the circulatory and respiratory systems, neoplasms, digestive system diseases, and infectious and parasitic diseases.

**Conclusion:**

The study highlights COVID-19’s impact on mortality, showing variations by year, age, gender, and disease.

**Contribution:**

Excess non-COVID-19 deaths likely stemmed from disrupted healthcare services. These findings underscore the need for ongoing monitoring of hospital mortality to identify pandemic-related service disruptions and guide interventions to strengthen healthcare services, improve access to care, and enhance referral systems during unexpected disasters.

## Introduction

The COVID-19 pandemic had a devastating impact on public health and healthcare systems during the period between 2020 and 2022.^1,2,3^ Before the pandemic, global health showed improvement and an overall steady decline in mortality trends.^4,5^ This trend was influenced by the global shift to address equity, health systems and the social determinants of health.^5^ Landmark developments include the adoption of the millennium development goals and later the sustainable development goals.^5^ These strategies have contributed to declines in exposure to risks and improved health.^6^ Over time, the global burden of disease patterns has transitioned towards more of the burden being because of non-communicable diseases and injuries. Notwithstanding these global shifts, South Africa still has a complex burden of diseases, characterised by a combination of communicable diseases, maternal and child health-related conditions, non-communicable diseases and injuries. Based on the findings of the South African Rapid Mortality Surveillance (RMS) for 2019 and 2020, the under-5 mortality rate was 28 per 1000 live births, with the maternal mortality rate (MMR) being 109 per 100 000 live births. The risk of a 30-year-old dying before 70 years of age from preventable non-communicable diseases (NCDs) was 32% for males and 23% for females.^7^

Currently, Statistics South Africa (Stats SA) compiles cause-of-death statistics based on death notifications but reports limited information at the district level. Nonetheless, this information still gives a valuable picture of mortality nationally.^8,9^ Mortality rates in SA began to decrease in the early 2010s mainly as a result of implementing HIV management programmes.^10^ The greatest impact on HIV-associated mortality has been the free provision of antiretroviral therapy (ART) since 2004 which reduced adult mortality to only a quarter of what it would have been in the absence of ART.^11^ By 2017–2018, prevention of vertical HIV transmission reduced infant HIV acquisition by 84% and HIV deaths in children by 94%, relative to what would have been expected without interventions.^12^ This downward mortality trend was severely interrupted by the COVID-19 pandemic, with deaths from April 2020 through March 2022 increasing significantly.^13^ Deaths because of all causes combined in 2020 and 2021 were in excess of what might have been expected without COVID-19.^14^

At both national and provincial levels in South Africa, excess mortality which refers to a rise in the number of deaths occurring above the expected level during a particular period, rose during the COVID-19 pandemic, with a demonstrable increase in COVID-19 deaths and a decline in non-COVID-19 deaths because of causes such as trauma and influenza during the strict lockdown period of early 2020.^14,15^ Reported COVID-19 deaths during the pandemic were skewed towards those known to have died from confirmed COVID-19 occurring within health facilities and likely underestimated COVID-19 deaths outside of medical facilities.^14^

Estimates of mortality and disease burden in South Africa largely rely on vital registration data, which are subject to time lags.^16^ Furthermore, few studies have used hospital-based data from the COVID-19 era for mortality surveillance. Hospital-level mortality data can serve as a lens to examine any variability in health system performance and disease contributing to mortality.^17^ Hospital mortality data can contribute to local understanding of disease burden variation and may also be key to monitoring the quality and coverage of routine health services provided both within the hospital and facilities referring to the hospital.^17^ Given the fluctuations in deaths and excess mortality nationally during the pandemic, it is of value to investigate the in-hospital patient mortality to assess the direct and indirect impact of COVID-19 at the facility level.

In South Africa, there is a tiered approach to categorising hospitals and an ability to refer up or down depending on the level of clinical care required.^18^ Academic hospitals consist of highly specialised national referral units that provide an environment for multi-speciality clinical services. These services cater to complex clinical cases and oftentimes require high technology and/or multi-disciplinary teams with scarce skills to provide care. Studies from low- and middle-income countries report a higher mortality rate in tertiary referral hospitals where patients with complex and/or multiple diseases are managed.^19,20,21^ Mortality rates at these hospitals are expected to be high in comparison to other hospitals and provide an indication of the types of conditions and diseases managed at these facilities, especially for those actively managing COVID-19 cases. Excess mortality among non-COVID-19 patients because of dislocation or interruption of standard care caused by resource prioritisation for COVID-19 has been reported globally.^22,23^ A cause-of-death analysis in the year preceding the COVID-19 pandemic could be established as a baseline for the impact of COVID-19 in subsequent years. Ongoing monitoring of mortality rates is expected to demonstrate any rebound in the pre-COVID causes and degree of mortality at the facility level. Our study aimed to investigate changing trends of hospital patient mortality in a hospital setting in Gauteng province, South Africa, from April 2018 through March 2022 representing pre-COVID-19 and COVID-19 periods.

## Research methods and design

### Study design

We conducted a retrospective record review of all patients who died in a hospital complex from 01 April 2018 to 31 March 2022.

### Study site

The study was conducted at the Steve Biko Academic (SBAH) and Tshwane District Hospital (TDH) complex, comprising two structurally independent hospitals, which function operationally as a continuous complex. The SBAH is an 830-bed academic hospital, located next to the 240-bed district hospital. The SBAH caters mainly to complex, comprehensive tertiary services, while TDH delivers level-one district hospital services. However, during the pandemic, the hospitals functioned as a single complex for the management of COVID-19. At the peak of the pandemic, the district hospital was repurposed for COVID-19 management to include intensive care unit (ICU), high-care and general COVID-19 beds, while the academic tertiary hospital provided all other services. Over the course of the subsequent COVID-19 waves, the hospitals were constantly reconfigured, and services were redistributed for both COVID-19 and general services.

### Study population

Patients who were admitted and subsequently demised in the hospital complex during the study period were included in this study. All deaths during the study period were considered for analysis, including all age groups and all causes of mortality. Patients who were admitted before 01 April 2018 and died in that period were included in the analysis. Patients admitted in the period and who died after 31 March 2022 were excluded from the analysis.

### Data collection

In the SBAH, deaths of admitted patients are routinely classified with ICD10 codes by clinicians and submitted to the Health Information Office (HIS). The HIS reports were used to obtain cases to include in this study. Data collected included patient file number, name, date of birth, gender, admission and death dates, ward and ICD10 code. For missing or incomplete data, details were extracted from mortuary registers (TPH 205) and patient records obtained from the hospital’s records department. Conversely, the district hospital lacked a routine system, so data were extracted from mortuary registers and supplemented with patient records when necessary. Collected data included file number, name, date of birth, age, death date, ward and ICD10 cause of death. The principal investigator, assisted by two research assistants, collected the data. Admission data for the period 01 April 2018 to 31 March 2022 for both hospitals were obtained from the Patient Administration and Billing System (PAAB). All data were imported into R software for analysis.

### Data analysis and management

Death trends were analysed according to the Department of Health financial years (April–March). Patient data were de-identified. Descriptive statistics analysed socio-demographic data, with age described by mean and standard deviation. The mortality rate was calculated using admissions as a denominator. Categorical variables, such as causes of death, were described using frequencies and percentages. The leading causes of death and the top three subcategories were reported as frequencies and percentages using ICD10 coding classification. Chi-squared tests assessed the significance of deaths by year, age group, and gender and a correlation between these variables was reported. Age categories were created and adapted from the age data reported by Statistics SA for mortality and causes of death notifications.^24^ Data analysis was conducted using R software.

### Ethical considerations

Ethics approval was granted by the University of Pretoria Ethics Committee on 31 March 2022 (reference no.: 54/2022). Permission to use routinely collected hospital data was obtained from hospital management.

## Results

**FIGURE 1:**
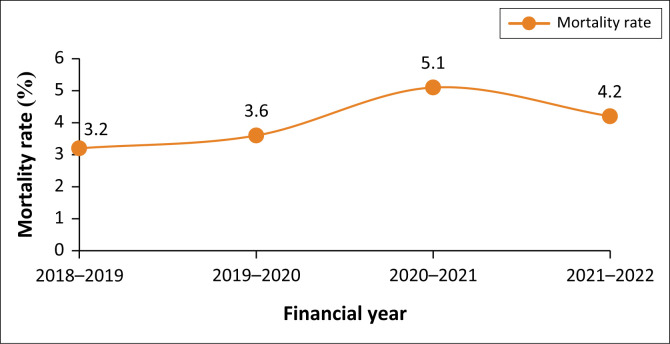
Mortality rate over time showing peak mortality in 2020–2021.

In the 4-year period under review, 7815 deaths were reported in the hospital complex. Of these, 32 were dead on arrival. The mortality rate is shown in Table 1 and Figure 1. The mortality rate increased over the period, with a lower baseline of 3.2% in 2018–2019, peaking at 5.1% in 2020–2021 and decreasing to 4.2% in 2021–2022, which is slightly above the pre-COVID-19 baseline. Noting that while the number of admissions in 2020–2021 decreased, the number of deaths remained constant. This difference directly influenced the mortality rate, reflecting a higher proportion of deaths relative to admissions.

**TABLE 1:** Trends in mortality rate (2018–2022).

Year	Admissions (*n*)	Deaths (*n*)	Mortality rate (%)
2018–2019 Pre-COVID-19	58 148	1853	3.2
2019–2020 Pre-COVID-19	58 114	2092	3.6
2020–2021 COVID-19 waves 1 and 2	35 956	1846	5.1
2021–2022 COVID-19 waves 3 and 4	48 259	2024	4.2

**Total**	**200 477**	**7815**	**3.9**

COVID-19, coronavirus disease 2019.

### Demographic characteristics

**FIGURE 2:**
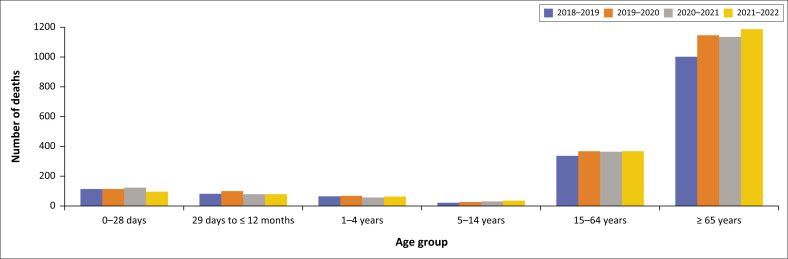
Deaths by year and age group.

**FIGURE 3:**
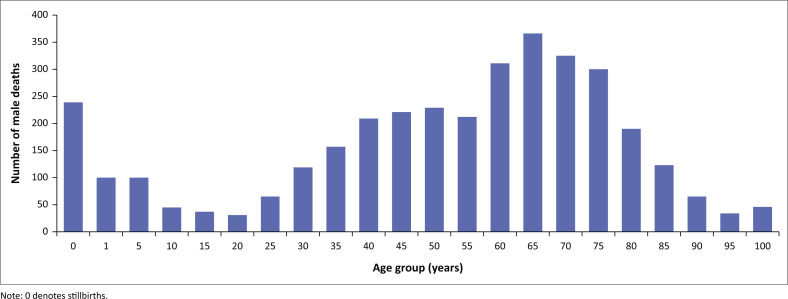
Distribution of male deaths by age from 2018 to 2022.

**FIGURE 4:**
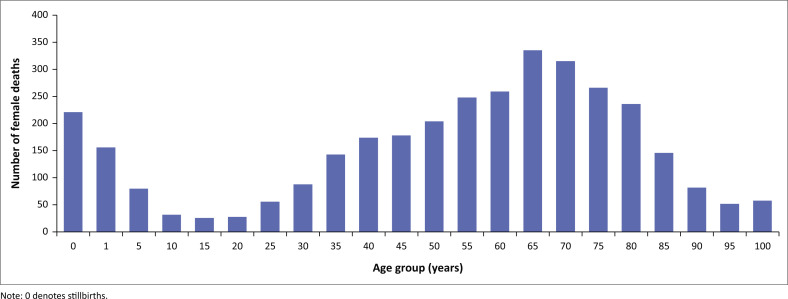
Distribution of female deaths by age from 2018 to 2022.

The demographic characteristics are summarised in Table 2, Figure 2, Figure 3 and Figure 4. The median age at death was 55.0 years (IQR 34–68). The analysis of mortality trends reveals distinct patterns in the different age groups with bimodal distributions in two extremes in age groups (under 5 and above 65 years). There were 41 stillbirths in the 4-year period. As illustrated in Figure 3 and Figure 4, deaths in children under 5 years accounted for 12.3% (955/7774) of total deaths. Neonates contributed to 47.8% (457/955) of under 5 years deaths excluding stillbirths. Thereafter, there was a decline in mortality with the 11–20 year age group having the lowest mortality, as seen in Figure 3 and Figure 4. From age 25 years upwards, for both males and females, there was a gradual increase in mortality, peaking around the 65-year age group. Thereafter, a gradual decline in deaths is observed in the older age categories. Among individuals older than 5 years, the highest mortality rate was observed in 2020–2021.

**TABLE 2:** Annual death distribution by age group and gender (2018–2022).

Age groups	2018–2019 deaths (*N* = 1853)		2019–2020 deaths (*N* = 2092)		2020–2021 deaths (*N* = 1846)		2021–2022 deaths (*N* = 2024)		Male deaths (*N* = 3946)		Female deaths (*N* = 3652)		Unknown gender (*N* = 217)	
	*n*	%	*n*	%	*n*	%	*n*	%	*n*	%	*n*	%	*n*	%
Stillbirths	15	0.8	17	0.8	3	0.2	6	0.3	2	0.1	12	0.3	27	65.9
0–28 days	112	6.3	130	6.3	123	6.8	92	4.6	206	5.6	250	6.3	1	0.2
29 days to ≤ 12 months	72	3.9	82	3.9	81	4.4	86	4.2	166	4.5	155	3.9	0	0.0
1–4 years	58	3.1	38	1.8	37	2.0	44	2.2	79	2.2	97	2.5	1	0.6
5–14 years	34	1.8	46	2.2	31	1.7	36	1.8	59	1.6	87	2.2	1	0.7
15–64 years	870	47.0	971	46.4	984	53.3	974	48.1	1760	48.2	1984	50.3	55	1.4
≥ 65 years	485	26.1	554	26.5	540	29.3	601	29.7	1121	30.7	1022	25.9	37	1.7
Unknown age group	207	10.9	254	12.1	47	2.4	185	9.1	259	7.1	339	8.6	95	13.7

**FIGURE 5:**
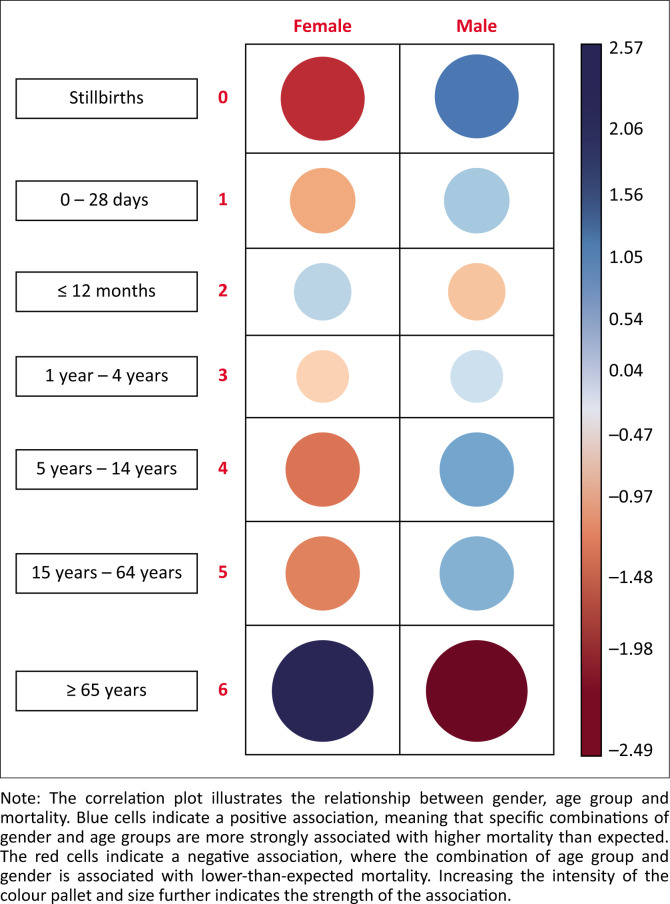
Balloon correlation plot on the effect of gender and age group on mortality.

There were more deaths among males compared to females, with the largest gender differences occurring in the higher age categories as shown in Figure 5. However, females in the age category ≥ 65 years experienced disproportionately higher mortality. The greatest difference in mortality by gender was in 2018–2019 and the least difference by gender during the peak COVID-19 pandemic, in 2019–2020 and 2020–2021.

### Cause of mortality

Table 3 shows the causes of mortality among patients based on the broad ICD-10 classification. The cause of death was unknown for 0.8% (63) of the sample. The top five broad causes of mortality were related to diseases of the circulatory system, respiratory system, neoplasms, digestive system and infectious and parasitic diseases. The leading causes of death at the start of the study period were diseases of the circulatory system, declining from 25.4% of deaths in 2018–2019 to 16.3% in 2021–2022. Conversely, mortality because of respiratory system diseases increased from 13.3% in 2018–2019 to 31.2% in 2021–2022, consistent with the evolution of the COVID-19 pandemic. Deaths because of diseases of the digestive system remained stable, while deaths because of neoplasms showed a slight increase in the first year of the pandemic, peaking in 2020–2021 at the height of the COVID-19 pandemic.

**TABLE 3:** The overall distribution of causes of death by ICD-10 main group cause of death.

Disease diagnostic group (ICD-10-code)	2018–2019		2019–2020		2020–2021		2021–2022		Total
	*n*	%	*n*	%	*n*	%	*n*	%	
Certain conditions originating in the perinatal period	96	5.2	94	4.5	69	3.7	53	2.6	312
Certain infectious and parasitic diseases‡	146	7.9	231	11.0	139	7.5	104	5.1	620
Codes for special purposes†	2	0.1	4	0.2	-	0.0	5	0.2	11
Congenital malformations	59	3.2	60	2.9	62	3.4	70	3.5	251
Diseases of the blood and immune mechanism	18	1.0	23	1.1	10	0.5	11	0.5	62
Diseases of the circulatory system‡	472	25.4	400	19.1	346	18.7	330	16.3	1548
Diseases of the digestive system‡	108	5.8	120	5.7	95	5.1	107	5.3	430
Diseases of the ear and mastoid process	1	0.1	-	0.0	-	0.0	-	0.0	1
Diseases of the eye and adnexa	3	0.2	1	0.0	1	0.1	1	0.0	6
Diseases of the genitourinary system	81	4.4	135	6.5	69	3.7	70	3.5	355
Diseases of the musculoskeletal system	15	0.8	23	1.1	18	1.0	24	1.2	80
Diseases of the nervous system	86	4.7	93	4.4	73	4.0	74	3.7	326
Diseases of the respiratory system‡	247	13.3	309	14.8	465	25.1	631	31.2	1652
Diseases of the skin and subcutaneous tissue	7	0.4	12	0.6	3	0.2	5	0.3	27
Endocrine, nutritional and metabolic diseases	42	2.3	48	2.3	46	2.5	27	1.3	163
Factors influencing health status and contact with health services	3	0.2	9	0.4	16	0.9	8	0.4	36
Injury, poisoning and certain other consequences of external causes	81	4.4	71	3.4	71	3.8	94	4.6	317
Mental and behavioural disorders	2	0.1	6	0.3	6	0.3	5	0.2	19
Neoplasms‡	290	15.7	346	16.5	293	15.9	271	13.4	1200
Other ill-defined and unspecified causes of mortality	0	0.0	1	0.0	-	0.0	-	0.0	1
Pregnancy, childbirth and puerperium	4	0.2	7	0.3	1	0.1	3	0.1	15
Symptoms and signs not elsewhere classified	79	4.3	80	3.8	53	2.9	108	5.3	320
Unknown	11	0.6	19	0.9	11	0.6	22	1.1	63

**Grand total**	**1853**	**100.0**	**2092**	**100.0**	**1846**	**100.0**	**2024**	**100.0**	**7815**

† , Codes in this range are used for provisional assignment where aetiology is uncertain;

‡ , The top five broad causes of mortality throughout the study period.

The differences in leading causes of death were similar among males and females. More males died because of diseases of the circulatory and respiratory system than women. Conversely, a greater proportion of women died because of neoplasms. There was a significant difference in the contribution of natural and unnatural causes of death to overall mortality by gender, with males contributing more to deaths from unnatural causes (*p* < 0.001). These data are shown in Table 4.

**TABLE 4:** Deaths by leading disease group and gender.

Disease diagnostic group (ICD-10-code)	Unknown gender		Female deaths		Male deaths		Grand total
	*n*	%	*n*	%	*n*	%	
Diseases of the circulatory system	25	2	724	47	799	51	1548
Diseases of the respiratory system	93	6	731	44	827	50	1651
Neoplasms	5	1	636	53	559	46	1200
Certain infectious and parasitic diseases	21	3	253	41	346	56	620
Diseases of the digestive system	6	1	215	50	209	49	430

Table 5 shows further sub-analysis by direct cause of death per broad disease category, limited to the top three disease categories. Mortality because of cardio-pulmonary arrest, congestive cardiac failure and cerebrovascular accident together were responsible for almost half (761/1548; 49.2%) of the deaths in the diseases of the circulatory system category. Although COVID-19 was reported only in two of the financial years of the study period, it still accounted for a significant proportion (627/1652; 38.0%) of deaths in the diseases of the respiratory system category overall. In the neoplasms category, female cancers dominate, with cervical and breast cancer being responsible for (210/1200; 17.5%) all cancer-related deaths. In the infectious and parasitic disease category, sepsis accounted for the greatest proportion of deaths (265/620; 42.7%) followed by deaths because of tuberculosis (205/620; 33.0%). Human immunodeficiency virus reported deaths were few (9/620; 1.5%). Gastroenteritis was the third leading cause of death in the category, and a consistent decline in the number of deaths is noted. In the category of diseases of the digestive system, liver diseases accounted for the greatest proportion of deaths (110/430; 25.6%).

**TABLE 5:** Top three sub-disease diagnostic groups per broad (ICD-10-code) category.

Top three sub-disease diagnostic groups per broad (ICD-10-code) category	2018–2019	2019–2020	2020–2021	2021–2022	Grand total	
	
	*n*	*n*	*n*	*n*	*N*	%
**Diseases of the circulatory system (I00–I99)**	**-**	**-**	**-**	**-**	**1548**	**19.8**
Cerebrovascular accident (I63–I64)	48	36	35	32	151	10.0
Congestive cardiac failure (I50)	57	66	57	76	256	16.0
Cardio-pulmonary arrest (I46)	173	96	32	60	361	23.0
**Diseases of the respiratory system (J00–J99)**	**-**	**-**	**-**	**-**	**1652**	**21.1**
Chronic obstructive pulmonary disease (J44)	39	71	29	19	158	9.5
Pneumonia (J18)	107	134	61	51	353	21.0
COVID-19 (U07 and J12.8)	-	-	213	414	627	38.0
Neoplasms (C00–D48)	-	-	-	-	1200	15.3
**Acute lymphocytic leukaemia (C91)**	**18**	**15**	**22**	**17**	**72**	**6.0**
Breast (C50)	23	20	26	25	94	8.0
Cervix (C53 and D06.9)	22	31	36	27	116	10.0
**Certain infectious and parasitic diseases (A00–B99)**	**-**	**-**	**-**	**-**	**620**	**7.9**
Gastro-enteritis (A09)	13	7	2	2	24	4.0
Tuberculosis (A15–A19)	45	100	35	25	205	33.0
Sepsis (A40–41)	70	80	56	59	265	42.0
**Diseases of the digestive system (K00–K95)**	**-**	**-**	**-**	**-**	**430**	**5.5**
Gastro-intestinal haemorrhage (K92)	16	13	13	16	58	13.0
Bowel obstruction (K56)	13	14	16	13	56	13.0
Liver disease (K70–K77)	32	35	24	19	110	25.0

The leading causes of death among children and adolescents (birth – 19 years) vary by age group. Conditions arising from prenatal (stillbirth) and early neonatal (from birth to 7 days) periods account for the majority of deaths in the perinatal category (312; 64.0%), while congenital malformations are the leading cause of mortality in infants under 1 year of age (109/331; 32.9%). Notably, neoplasms emerge as a substantial cause of mortality in the 10–19 age group (35; 28.0%). Certain infectious and parasitic diseases contribute significantly to all deaths from the perinatal period through to adolescence (115;10.0%).

## Discussion

This study looked at the mortality rate over 4 years in a hospital complex of the public health sector consisting of a central hospital and a district hospital in South Africa, offering a glimpse into the mortality trends and causes of death in that hospital complex prior to and during the COVID-19 pandemic. This study reports a significant increase in the mortality rate from 3.3% to 4.2% in the study period, with the highest mortality rate observed at 5.1% during the onset of the COVID-19 pandemic (2020–2021). The slightly lower mortality rate in the 2021–2022 year can be attributed to the decreased severity of disease of the dominant SARS-CoV-2 Omicron variant in the fourth COVID wave from December 2021 through February 2022.^25^ Although COVID-19 was reported only in two of the financial years of the study period, it still accounted for a significant proportion (37%) of deaths in the diseases of the respiratory system category overall.

The median age at death was 55 years (IQR 34–68). Deaths in children under 5 years accounted for 12.8% of all deaths. The observed decline in the under-5 deaths is consistent with the population-based studies.^26^

During the study period, most deaths occurred in the age category 15–64 years in both males and females, in keeping with the higher admissions in this age group. A steady increase in death trends among patients older than 65 years was noted. During the period of review, there were more male deaths than female deaths, consistent with other studies in the African region as well as South Africa.^26,27^ However, it is interesting to note the narrowing gap between female and male deaths during the pandemic. Dorrington and colleagues postulated the reason for this difference in mortality by gender in 2020 is a result of the higher number of females surviving to old age, and thus being more vulnerable to COVID-19 and related mortality.^7^

This study found the top five broad causes of mortality were related to diseases of the circulatory system, respiratory system, neoplasms and diseases of the digestive system, and infectious and parasitic diseases. The findings of this study differ from pre-COVID-19 South African population-based mortality estimates, where HIV and diabetes were more prominent causes of death.^4^ The COVID-19 pandemic may have had a significant negative impact on the reporting of deaths primarily because of HIV. Additionally, the difference may be attributed to the institutional setting of the study (academic hospital influence) and differences from population-level data. Lastly, the low reported rate of HIV-related deaths may also stem from the underreporting of HIV/AIDS as a direct cause and the overall decline in HIV-related mortality because of widespread access to ART.^28,29^ With ART significantly improving life expectancy, the primary causes of death among HIV-infected individuals have shifted, with cardiovascular diseases, non-AIDS-defining cancers and liver conditions now being the leading contributors to mortality.^28,30,31^ Deaths because of diseases of the respiratory system increased in the same period causing 13.3% of all deaths in the year 2018–2019 as compared to 31.2% in the year 2021–2022, consistent with the waves of the COVID-19 pandemic. Deaths because of diseases of the digestive system remained stable, while deaths because of neoplasms showed fluctuation, peaking in years 2019–2020 and 2020–2021 at the height of the COVID-19 pandemic, possibly because of lack of services or difficult access to services, and individual financial difficulties during the outbreak were affecting health-seeking behaviour.^32^ On the supply side, the survey identified the cancellation of elective care and the redeployment of staff to strengthen the response to COVID-19 as contributing factors.^32^ Similar measures had been taken by the government and across hospitals, both in public and private healthcare sectors in South Africa in response to the pandemic. Mortality because of cardio-pulmonary arrest, congestive cardiac failure and cerebrovascular accident together were responsible for almost half (48%) of the deaths in the diseases of the circulatory system category. This is consistent with the increase in the prevalence of non-communicable diseases in sub-Saharan Africa, influenced by lifestyle and behaviour.^33^ In addition, it may also be associated with delayed referrals and presentation to healthcare facilities and warrants further investigations.

This study identified neoplasms as one of the leading causes of death (reported 9th in other reports).^4^ The predominance of female cancers, particularly cervical and breast cancer (210 deaths; 18% of all cancer-related deaths), underscores the need for effective primary and secondary prevention programmes. Additionally, while the mortality trend over the short term in this study showed a slight increase, surgical delays and delayed access to cancer treatment may negatively impact long-term survival rates, highlighting the need for continued monitoring.^34^ In the infectious and parasitic disease category, sepsis accounted for the greatest proportion of deaths (265; 42%). This was followed by deaths because of tuberculosis (205; 28%), which is of major concern because of the preventable and treatable nature of the disease, and the commitment from the South African Department of Health to reduce TB deaths in alignment with the WHO 2030 goals.^35^ While gastroenteritis was the third leading cause of death in the category, a consistent decline in the number of deaths is noted. In the category of diseases of the digestive system, liver diseases accounted for the greatest proportion of deaths (110; 24%).

The primary causes of death in 0–20 years age groups in this study largely follow reported trends: perinatal conditions are the leading cause in the age group covering stillbirths and early neonatal deaths (perinatal period), congenital malformations contribute to most deaths in infants (29 days to less than 1 year) and neoplasms become more prominent in adolescents aged 10–19 years, with infectious and parasitic diseases affecting all age groups.

The dual burden of infectious and non-communicable diseases is common in the African region. Kalyesubula et al. published findings on trends of mortality rates among medical in-patients at a tertiary hospital in Uganda in 2019, highlighting similarly the high burden of hypertension, stroke and cancer, as well as non-TB pneumonia.^36^ Our study reported significantly lower deaths because of HIV and a declining number of TB deaths. Additionally, the period under review in our study refers to health systems functioning and access to care as influenced by the COVID-19 national response which led to delays in accessing healthcare, and interruptions to routine and emergency services. In the South African context, Pillay et al. examined the impact of COVID-19 on routine public sector PHC services in South Africa between 2019 and 2021. They reported a decrease in screening and laboratory testing for both HIV and TB during the COVID-19 pandemic, suggesting a negative impact of COVID-19 on non-COVID-19 testing and treatment services.^7^ Burger et al. in their South African-based study demonstrated that 4% of uninsured patients who needed chronic care during the COVID-19 pandemic, did not seek care.^37^ In their analysis of routine health system data, they found that a very large share of unmet healthcare needs can be attributed to COVID-19, indicating that the pandemic has had considerable unintended public health consequences.^38^

Mortality trends by cause of death provide valuable insights into healthcare needs, such as the necessity for preventive measures like cancer screening, early diagnosis programmes for chronic diseases, access to treatment for infectious diseases and palliative care services for terminal illnesses. Analysis of mortality trends enables efficient resource prioritisation by identifying emerging health needs and vulnerabilities within the healthcare system, guiding targeted interventions to enhance resilience including the ability to be flexible and adaptable in the delivery of healthcare services. Additionally, cause-specific mortality data support evidence-based health system responses to address infectious and non-communicable diseases while providing a basis to evaluate the effectiveness of pandemic response strategies and recovery efforts. Lastly, cause-specific mortality data provide an opportunity to develop interventions required to strengthen pandemic-affected services, interrupted access to care and referral systems.

### Limitations

Causes of death were based on clinician-reported primary causes, which are subject to clinician experience, and do not reflect additional underlying conditions. The study assumes that reasonable accuracy was maintained for the allocation of ICD10 codes in hospital records. Incomplete data resulted from incomplete reporting or unavailable information for patients dead on arrival with limited demographic details available. Records without a reported cause of death were categorised as unknown deaths. The study is limited by its focus on two hospitals in a single province. Despite these limitations, it is one of the few studies analysing causes of death at the facility level before and during COVID-19. It provides data on causes of death, enabling targeted health system interventions and quality improvement. Population-based mortality analyses are dependent on vital registration data, which are subject to time lags in their release. This study is not constrained by the time lag in the release of vital registration data, providing an opportunity for the facilities studied to respond promptly to changes in mortality and address healthcare access and services.

## Conclusion

In the wake of the COVID-19 pandemic and the post-COVID recovery phase, sound evidence on mortality trends by cause of death is essential for health system strengthening. This hospital-based study confirmed the increase in mortality during COVID-19 and the slow decline in mortality after the peak of the pandemic, without returning to the baseline before COVID-19. While the overall mortality rate increase to higher than before the COVID-19 pandemic should be interpreted cautiously, we believe the higher rate warrants ongoing analysis of mortality trends. Our study highlighted the contribution of both communicable and non-communicable diseases in mortality in a region in South Africa, as well as differences by gender and age group. It also identified the impact of COVID-19 on hospital-based mortality. The observed excess mortality highlights the significant strain placed on healthcare services during and after the peak of the COVID-19 pandemic. This increase is likely because of a combination of factors, including COVID-19-related deaths, delays in accessing healthcare, interruptions to routine and emergency services and the compounding effects of untreated or poorly managed non-COVID-19 conditions. The findings of this study can be used as guidance to identify acute and chronic disease screening and treatment programmes impacted negatively by the pandemic resulting in an increase in mortality. Our recommendations include a focus on the allocation of resources to address the health service needs post-pandemic and identifying service vulnerabilities to enable the development of service continuity strategies during a crisis. We intend to conduct further sub-analyses to identify more specific nuances in the in-hospital mortality trends. We further recommend investment in capacitating health systems with critical capacities for mortality surveillance at sub-national levels. Lastly, we propose hospital-based capacity development programmes to strengthen the response to future pandemics while maintaining routine health services and disease control programmes.
